# Cortical neurons develop a senescence-like phenotype promoted by dysfunctional autophagy

**DOI:** 10.18632/aging.102181

**Published:** 2019-08-30

**Authors:** Daniel Moreno-Blas, Elisa Gorostieta-Salas, Alexander Pommer-Alba, Gabriel Muciño-Hernández, Cristian Gerónimo-Olvera, Luis Angel Maciel-Barón, Mina Konigsberg, Lourdes Massieu, Susana Castro-Obregón

**Affiliations:** 1 Departamento de Neurodesarrollo y Fisiología, División de Neurociencias, Instituto de Fisiología Celular, UNAM, Mexico City 04510, México; 2 Departamento de Neuropatología, División de Neurociencias, Instituto de Fisiología Celular, UNAM, Mexico City 04510, México; 3 Departamento de Ciencias de la Salud, Universidad Autónoma Metropolitana, Unidad Iztapalapa, Mexico City 09340, México

**Keywords:** senescence, brain, neuron, autophagy, SASP (senescence-associated secretory phenotype)

## Abstract

Senescent cells accumulate in various tissues and organs with aging altering surrounding tissue due to an active secretome, and at least in mice their elimination extends healthy lifespan and ameliorates several chronic diseases. Whether all cell types senesce, including post-mitotic cells, has been poorly described mainly because cellular senescence was defined as a permanent cell cycle arrest. Nevertheless, neurons with features of senescence have been described in old rodent and human brains. In this study we characterized an *in vitro* model useful to study the molecular basis of senescence of primary rat cortical cells that recapitulates senescent features described in brain aging. We found that in long-term cultures, rat primary cortical neurons displayed features of cellular senescence before glial cells did, and developed a functional senescence-associated secretory phenotype able to induce paracrine premature senescence of mouse embryonic fibroblasts but proliferation of rat glial cells. Functional autophagy seems to prevent neuronal senescence, as we observed an autophagic flux reduction in senescent neurons both *in vitro* and *in vivo*, and autophagy impairment induced cortical cell senescence while autophagy stimulation inhibited it. Our findings suggest that aging-associated dysfunctional autophagy contributes to senescence transition also in neuronal cells.

## Introduction

Aging is accompanied by a wide range of symptoms that reduce health span, such as cardiovascular dysfunction, osteoporosis, neurodegeneration and cancer, among other diseases. Amelioration of those symptoms has been achieved after pharmacological interventions using a novel class of drugs termed senolytics. These compounds clear senescent cells that accumulate late in life both in normal tissues and especially in those affected by age-related pathologies [1]. Nevertheless, senescent cells are harmful only when they persist, since transient senescent cells, which are eliminated by effector immune cells, contribute to wound healing, regeneration, immunity and even morphogenesis during development [2]. Therefore, it is fundamental to understand the molecular mechanisms of senescence establishment and maintenance.

Cellular senescence is a phenotype characterized by a durable cell cycle arrest (*i.e*. cells do not respond to mitogens) and a flattened and vacuolated morphology with stress granules collection. Even though there is no single marker of senescence, some of the most common features observed are: activation of the lysosomal enzyme senescence-associated β-galactosidase (SA-β-gal); persistent DNA damage response detected by γH2AX foci; expression of tumor suppressors p21^CIP1/WAF1^ (encoded by *Cdkn1a)* and/or p16^INK4A^ (encoded by *Cdkn2a)*; lipofuscin accumulation [3]; and nuclear deformation associated with nuclear envelop proteins degradation [4]. The most important activity of senescent cells is the secretion of a set of molecules, known as the senescence-associated secretory phenotype (SASP) that, depending on the physiological context, can be either beneficial or harmful. In early stages senescent cells secrete cytokines that promote the migration and infiltration of effector immune cells, as well as growth factors and proteases that facilitate tissue repair and remodeling. Yet, persisting signaling contributes to chronic inflammation, a hallmark of aging and a major contributor to age-related dysfunctions. SASP molecules also have an autocrine role, fostering the senescent phenotype, and a paracrine role inducing senescence in surrounding cells [5], inflammation and tumorigenesis [6,7].

Autophagy is a catabolic process that degrades intracellular components, like proteins and damaged organelles, including mitochondria, through lysosomes. Similar to senescence, it is induced in response to stressful stimuli, therefore both senescence and autophagy are often observed simultaneously. The interplay between autophagy and senescence requires further investigation, since autophagy has been implicated in both promoting and inhibiting cellular senescence. While autophagy inhibition promotes senescence in normal proliferating cells [8], autophagy inhibition delays oncogene-induced senescence and the synthesis of SASP components [9]. A possible explanation for the opposing role of autophagy over senescence is that it could engulf alternative targets that regulate cellular senescence in opposite manners. For example, the stability of the transcription factor GATA4, a key activator of SASP genes, is regulated by autophagy. Upon senescence induction, GATA4 escapes autophagic degradation because its interaction with p62/SQSTM1 (an autophagy cargo receptor) decreases, leading to GATA4 accumulation [10]. On the other hand, during oncogene-induced senescence, autophagy fosters the SASP through a specialized compartment known as the TOR-autophagy spatial coupling compartment (TASCC), where mTOR localizes at the surface of autolysosomes, which are surrounded by endoplasmic reticulum; a flux of recycled amino acids and metabolites released from the autolysosomes are used by mTORC1 for supporting the synthesis of SASP factors, hence facilitating senescence [11]. Therefore, selective autophagy actively suppresses cellular senescence through the degradation of GATA4, whereas autophagic degradation of other proteins (perhaps long-lived proteins), facilitates the SASP [12].

The traditional view of senescence as a specific phenomenon where a proliferation-competent cell undergoes permanent growth arrest, has limited the study of senescence of post-mitotic cells. Accordingly, the limited studies on cellular senescence in the brain have mostly focused on glial cells [13]. Nonetheless, some senescent markers have been described in several studies of both physiological aging and neurodegenerative diseases [14]. For example, cortical and Purkinje neurons show several senescence features like SA-β-gal activity, lipofuscin accumulation, γH2AX and macro-H2A foci, and IL6 expression, all in a p21^CIP1/WAF1^-dependent manner [15]. Interestingly, human neurons might also senesce, since there is expression of *p16/Cdkn2a* in pyramidal neurons in the prefrontal cortex from human brains of people over 77 years old [10]. We are interested in understanding the molecular basis for neuronal senescence, because we hypothesize that when senescent neurons persist in the brain, they contribute to cognitive decline by impairing synaptic function, inducing paracrine senescence and chronic inflammation.

While cellular senescence of mitotic cells is induced mainly by stressful stimuli (most of them inducing DNA damage), telomere attrition during cell division, oncogene activation or developmental cues [2], the molecular mechanisms that induce post-mitotic cells senescence are less understood. Also, whether autophagy regulates senescence in any direction in post-mitotic cells is completely unknown. Several groups have observed that neuronal cells acquire some senescent features *in vitro*, providing a very helpful system to study the molecular basis of neuronal senescence. For example, primary cortical, hippocampal and cerebellar granule neurons become SA-β-gal-positive over time [16–20]. However, these studies were limited to the detection of SA-β-gal activity, which could be misleading [21]. Recent *in vitro* studies confirmed the presence of additional senescent features, including γH2AX foci in neurons from mouse neuro-glial co-cultures maintained up to 27 days *in vitro* (D*IV*) [22]. Taken together, these reports support the notion that cultured neurons *in vitro* are capable of undergoing cellular senescence with the same features that occur *in vivo*.

For senescent neurons to contribute to chronic inflammation and paracrine senescence, they must establish a senescent phenotype including the SASP. Paracrine senescence has been demonstrated to occur in mouse embryonic fibroblast exposed to conditioned media from senescent fibroblasts [5]. In this work we developed an *in vitro* model of neuronal senescence that recapitulates *in vivo* senescence markers, and secreted molecules able to induce paracrine glial proliferation as well as premature senescence in mouse embryonic fibroblasts, pointing towards a neuronal SASP. We found that senescent cortical cells secrete C-C motif chemokine 2, also known as monocyte chemotactic protein 1 (MCP-1), a known SASP factor able to induce paracrine senescence [23]. Interestingly, we found that rat primary cortical neurons displayed features of cellular senescence before glial cells did. As reported for proliferating cells, functional autophagy, perhaps selective, seems to prevent neuronal senescence, as we observed autophagic flux impairment. Accordingly, we observed more senescent cortical cells when autophagy was impaired and less when it was stimulated. Our findings suggest that a dysfunctional autophagy contributes to senescence transition also in post-mitotic cells.

## RESULTS

### Primary cortical neurons acquire several senescent features after long-term culture.

**Figure 1:**
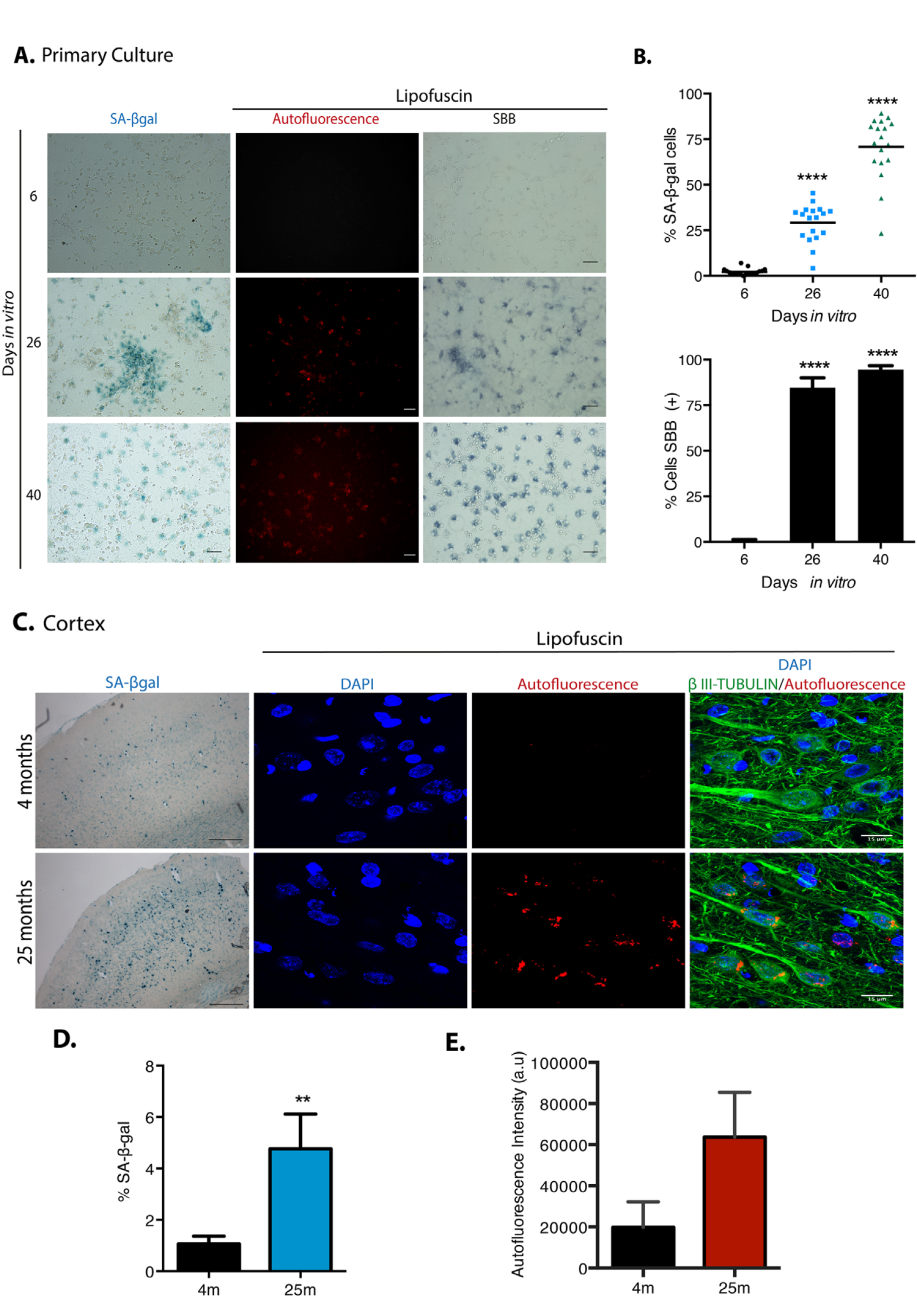
**Cortical cells in long-term culture and in old rat brains had higher SA-β-gal activity and accumulated lipofuscin.** (**A**) SA-β-gal activity or lipofuscin accumulation detected by autofluorescence or by Sudan Black B (SBB) staining were detected in primary rat cortical cells cultured for the indicated D*IV*. Notice that cortical cells have higher SA-β-gal activity and lipofuscin from 26 D*IV*. Images are representative of at least three independent experiments. Scales bar represent 100 μm. (**B**) Percentage of SA-β-gal or SBB positive cells in the cultures incubated at the indicated D*IV*. Quantification was made using NIS Elements software. The mean of three independent experiments, each done by quintupled replicas, is graphed. Bars in graphs represent SEM. Two-way RM ANOVA analysis, with Dunnett´s multiple comparison test. **** p< 0.0001 in comparison with 6 D*IV*. (**C**) Cortical neurons in old brains had higher SA-β-gal activity (scale bars represent 500 μm) and accumulated lipofuscin. Scale bar represents 15 μm. (**D**) The percentage of SA-β-gal positive area within each brain section is plot. The average of three brains per age is graphed; 15 sections from each brain were quantified. Bars in graphs represent SEM. Unpaired t Test, ** p< 0.01. (**E**) Quantification of autofluorescence intensity per section (arbitrary units). Bars in graphs represent SD. The average of three brains per age is graphed; 15 sections from each brain were quantified. Even though there was an evident increase in autofluorescence, no statistical significance was obtained.

To establish an *in vitro* model to study the transition of neurons from a non-dividing terminal differentiation state into senescence *in vitro*, prenatal rat cortical cells were cultured for up to 40 days *in vitro* (D*IV*). Since several reports indicate the presence of senescent glial cells in old brains [13], we considered that glial cells could become senescent and then promote paracrine neuronal senescence; hence, we allowed the proliferation of glial cells during the culture of primary cortical cells. During the first days of culture (6 D*IV*), neurons (expressing βIII-TUBULIN) represented 96.8% (SD 2.2) of the cells with very few glial cells (expressing GFAP); due to proliferation of glial cells and some loss of neurons, by 26 D*IV* there were 51% (SD 6.9) neurons and 31% (SD 11.5) glial cells; and by 40 D*IV* 73% (SD 6.9) of the surviving cells were glial. The cells expressing βIII-TUBULIN did not expressed GFAP. On average, the total number of cells along the culture remained similar (Supplementary Figure 1S). Without any further stressful stimuli, cortical cells became SA-β-gal-positive over time and accumulated lipofuscin detected by autofluorescence and by Sudan Black B staining, a lipophilic dye [24] (Figures 1A-B). SBB staining seems to be more sensitive than SA-β-gal activity. An increment in both SA-β-gal-positive and lipofuscin accumulation was also confirmed in the cortex of old rat brains (Figures 1C-E).

**Figure 2:**
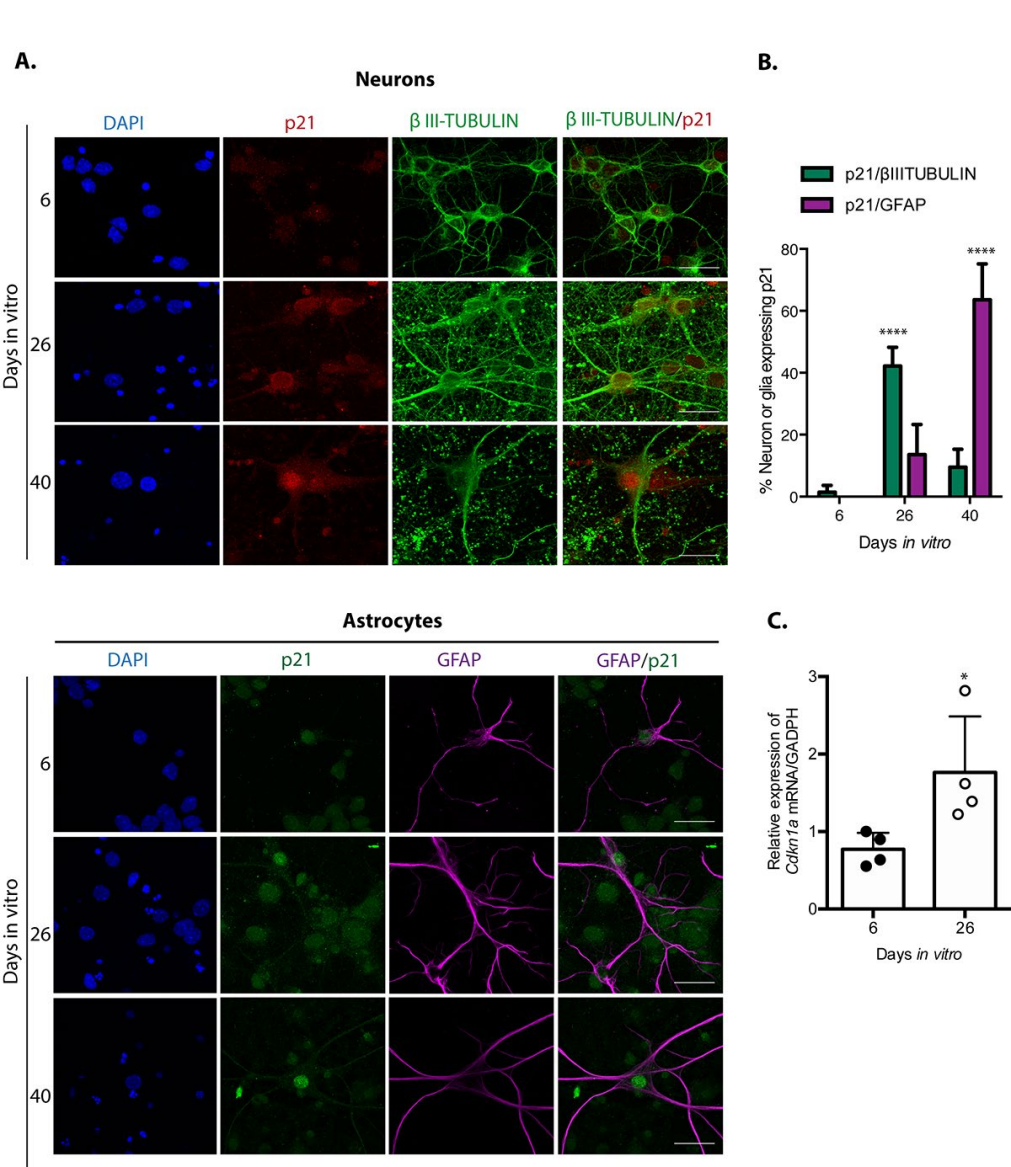
**Neuronal cells in cortical long-term culture showed increased expression of p21^CIP1/WAF1^.** (**A**) Immunofluorescence to detect p21^CIP1/WAF1^ (p21) in neurons (expressing βIII-TUBULIN) or astrocytes (expressing GFAP) in primary culture of cortical cells incubated during the indicated D*IV*. Notice that mostly neurons increased the abundance of p21^CIP1/WAF1^ at 26 D*IV*, indicating that neurons acquired senescent features before glial cells. Scale bar represents 25 μm. Arrows indicate examples of cells with healthy nuclei counted (not all the healthy cells are indicated). (**B**) Percentage of neurons or glial cells expressing p21^CIP1/WAF1^ over all cells. The mean of three independent experiments, each done by duplicate, is plotted. Bars represent standard deviation. Two-way RM ANOVA analysis, with Tukey´s multiple comparison test. **** p<0.0001. (**C**) qRT-PCR from total RNA purified from cortical primary cultures during the indicated days. The relative expression of *Cdkn1a* mRNA was normalized with *Gapdh* mRNA. Bars represent SD. * p=0.039 by unpaired t test two tailed. n=4.

Since different inducers of senescence in mitotic cells converge on the activation of the tumor suppressor p21^CIP1/WAF1^, and indeed p21^CIP1/WAF1^ has been suggested to mediate neuronal senescence in old brains [15], we reasoned that even though post-mitotic cells already express some CDK inhibitors to exit the cell cycle, they could still need to induce its expression for a pro-senescent activity from this protein. Therefore, we analyzed its expression on neurons or glial cells at different times of culture. As shown in Figure 2, in primary culture of cortical cells incubated 26 D*IV*, neurons but not yet glial cells expressed higher levels of p21^CIP1/WAF1^. The number of glial cells with elevated expression of p21^CIP1/WAF1^ increased until 40 D*IV*. This observation suggests that neurons acquire senescent features before glial cells. Interestingly, at 26 D*IV* p21^CIP1/WAF1^ is slightly enriched at the nuclear periphery. This could be related to the recent finding that altered nuclear export is a common hallmark of aging [25]. We confirmed p21^CIP1/WAF1^ expression is induced at transcriptional level in cortical cells at 26 D*IV* by qRT-PCR (Figure 2C).

**Figure 3:**
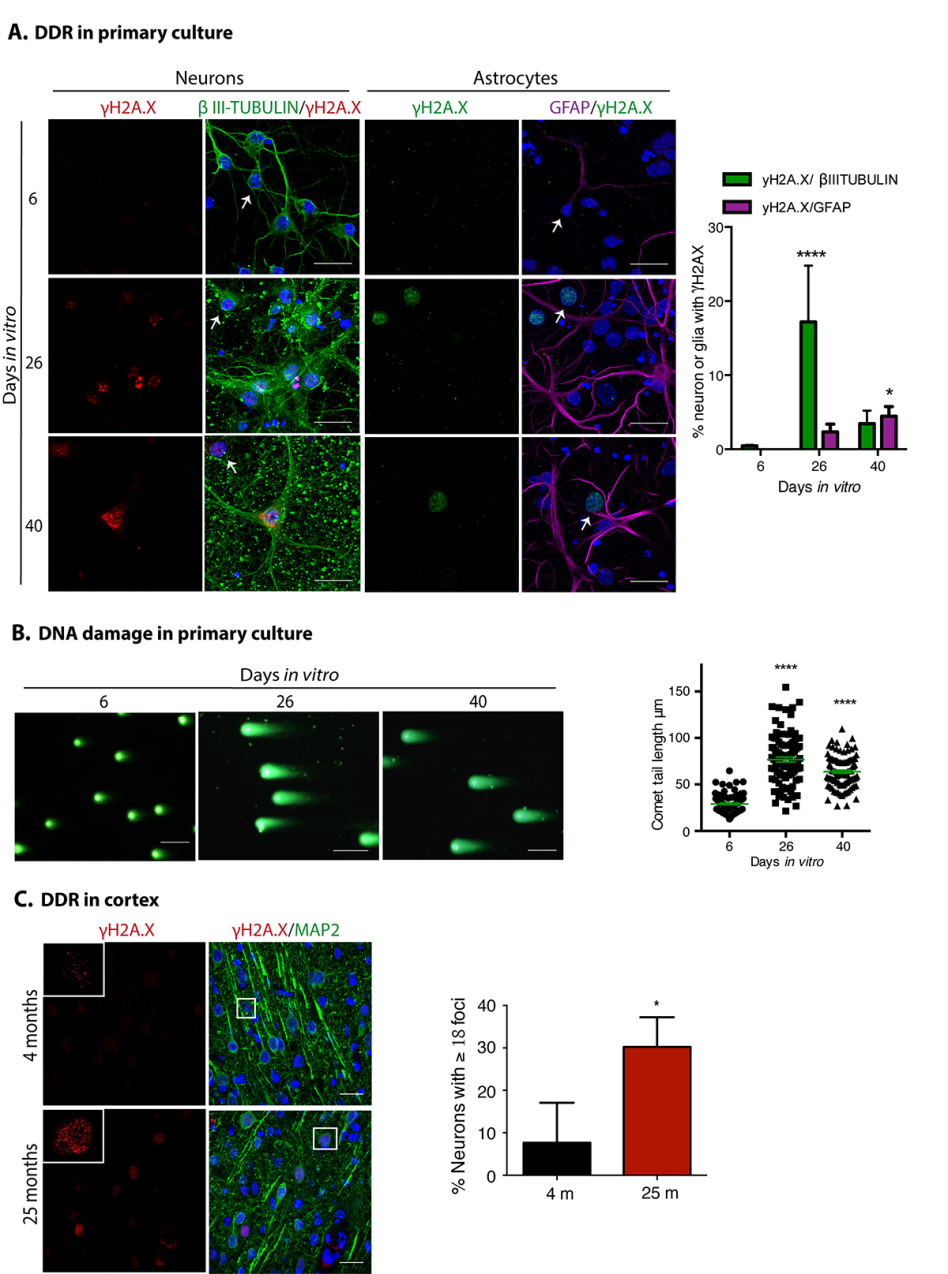
**Neuronal cells in cortical long-term culture and in the cortex from old rat brains had a sustained DNA damage response (DDR).** (**A**) Immunofluorescence to detect γH2AX foci in neurons (expressing βIII-TUBULIN) or astrocytes (expressing GFAP) in primary culture of cortical cells incubated during the indicated D*IV*. Notice that mostly neurons have γH2AX foci at 26 D*IV*. Scale bar represents 25 μm. Right, quantification of the percentage of neurons or glial cells with γH2AX foci over all cells. The mean of three independent experiments, each done by duplicate, is plotted. Bars represent SEM. Two-way RM ANOVA analysis, with Dunnett´s multiple comparison test. **** p< 0.0001 26 D*IV vs*. 6 D*IV*; * p<0.05 40 D*IV vs*. 6 D*IV*. Arrows indicate examples of cells with healthy nuclei counted (not all the healthy cells are indicated). (**B**) Comet assay to detect double strand breaks in genomic DNA from cells collected at the indicated days. Scale bars represent 100 μm. Right, the length of the tail of the comets, indicative of level of DNA damage, is plotted. 50 nuclei from each treatment, from two independent experiments, were analyzed by RM one-way ANOVA with Dennett’s´ multiple comparison. **** p< 0.0001 between 26 D*IV* or 40 D*IV* in comparison with 6 D*IV*. (**C**) Immunofluorescence to detect γH2AX foci in cortical neurons (expressing MAP2) in rat brains from the indicated age. Nuclei were stained with DAPI. Scale bars represent 30 μm. Right, percentage of neurons with more than 18 foci per nucleus. More than 100 neurons were counted from 3 different brains of each age. Bars represent standard deviation. Unpaired t Test * p<0.01.

Another hallmark of senescence is a persistent DNA damage response, commonly detected by the presence of γH2AX foci. As shown in Figure 3A, neuronal cells accumulated γH2AX foci at 26 D*IV*, accompanied by ruptures of DNA detected by Comet assay (Figure 3B). Even though we are not able to distinguish neurons from glial cells with this assay, it is conceivable that the nuclei with broken DNA come from neurons, since we observed that mainly neurons had γH2AX foci; these observations suggest that neurons accumulate DNA damage leading to a persistent DNA damage response. At both 26 and 40 D*IV*, only a small proportion of GFAP expressing cells had γH2AX foci. As expected, cortical neurons from old rat brains also contained more γH2AX foci (Figure 3C).

**Figure 4:**
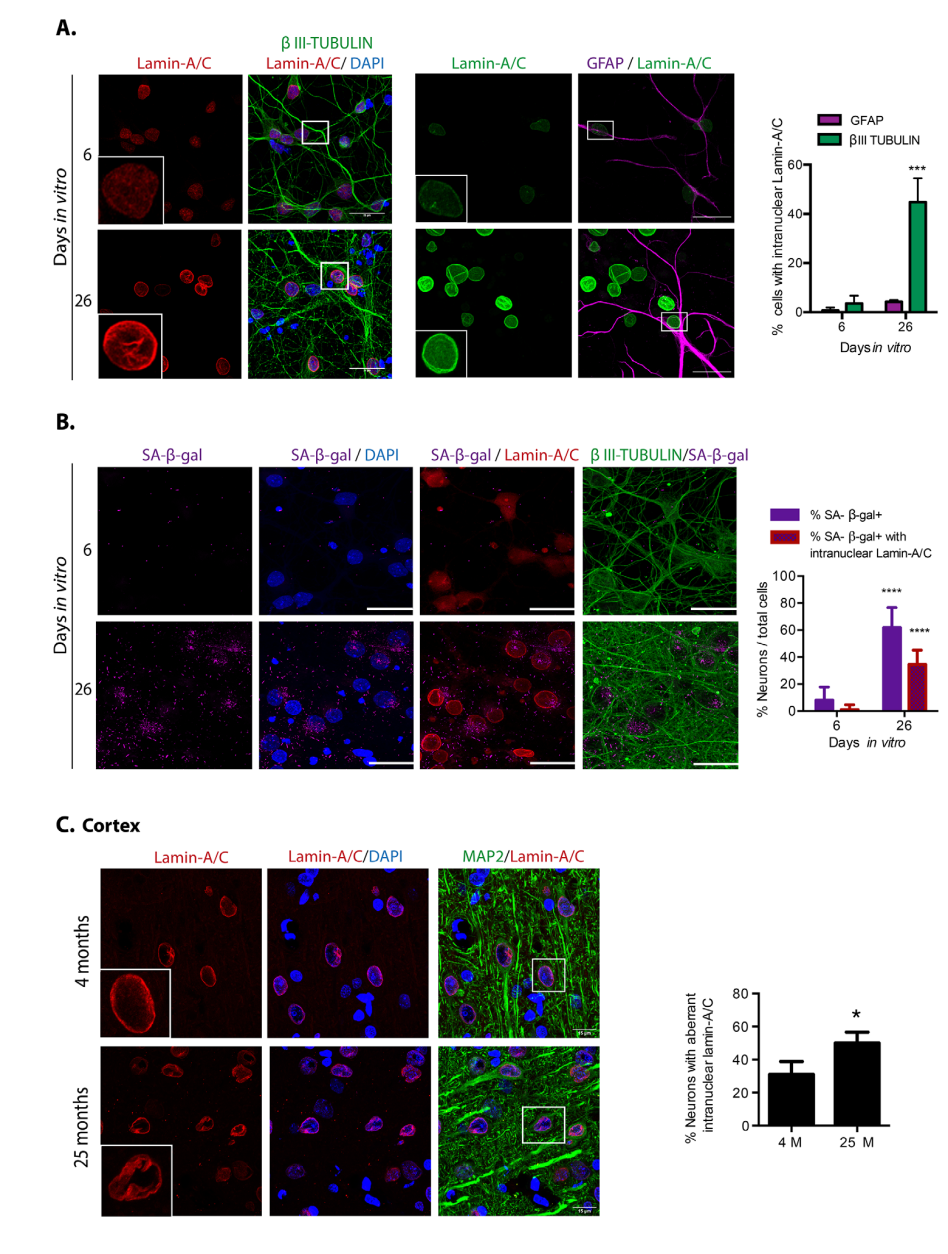
**Cortical cells in long-term culture and in old rat brains had nuclear morphology abnormalities.** (**A**) Immunofluorescence to detect Lamin-A/C in neurons (expressing βIII-TUBULIN) or astrocytes (expressing GFAP), in primary culture of cortical cells incubated during the indicated days *in vitro.* Squares indicate the magnified area shown in insets. Representative images of three independent experiments are shown. Scale bars represent 25 μm. Right, percentage of neurons or astrocytes with aberrant nuclear morphology over total cells. Bars represent SEM; two-way RM ANOVA analysis, *** p< 0.001 in comparison with 6 D*IV*. (**B**) Simultaneous detection of SA-β-gal activity (by confocal microscopy) and Lamina-A/C (by immunofluorescence) in neurons (expressing βIII-TUBULIN) in primary culture of cortical cells incubated during the indicated days *in vitro.* Representative images of three independent experiments are shown. Scale bars represent 25 μm. Right, percentage of neurons with visible SA-β-gal activity, and with both visible SA-β-gal activity and aberrant intranuclear Lamin-A/C over total cells. Five fields from three independent experiments were quantified. Bars represent SEM. Two-way RM ANOVA analysis, followed by Sidak´s multiple comparison test. **** p< 0.0001 in comparison with 6 D*IV*. C. Immunofluorescence to detect Lamin-A/C in cortical neurons in the internal pyramidal layer 5 from brain slices of the indicated age. Notice that also *in vivo,* neurons in old brains had nuclear deformations. Squares indicate the magnified area shown in insets. Scale bars represent 30 μm. Right, percentage of neurons in with aberrant nuclear morphology in cortical brain slices of the indicated age, as shown in (**C**). (n=3). Bars represent SD; unpaired t Test Student * p< 0.01.

Finally, since during oncogene-induced senescence, replicative senescence and senescence induced by DNA damaging drugs occur nuclear morphology abnormalities associated with nuclear envelope proteins loss [26], and depletion of Lamin-B1 or Lamin-A/C is sufficient to induce senescent features [4], we wondered whether also senescent post-mitotic cells, such as neurons, would manifest nuclear morphology deformations. As shown in Figure 4, indeed both *in vitro* and *in vivo* senescent neurons had irregular nuclear morphology with folds of the nuclear envelope forming intra-nuclear Lamin-A/C structures that protrude into the nucleoplasm. Very few astrocytes showed abnormal distribution of Lamin-A/C at 26 D*IV*, strengthening the notion that neurons become senescent before glial cells in this model. Since every senescent feature, alone, is not sufficient to confirm a senescent state, we simultaneously detected SA-β-gal activity and Lamin-A/C in neurons. As shown in Figure 4B, around half of the neurons with high SA-β-gal activity at 26 D*IV* also had an aberrant nuclear morphology, supporting the notion that neurons in long-term culture acquire senescent features. The observation that some neurons with high SA-β-gal show a normal nuclear morphology, is in agreement with previous observation that SA-β-gal activity alone is not a reliable marker of senescence [21]. We propose that nuclear deformations could be a more reliable marker for neuronal senescence. Since differentiated neurons express low levels of Lamin-B1 [27], we were unable to detect loss of Lamin-B1 in senescent neurons.

### Senescent cortical cells secrete molecules that induce premature paracrine senescence and glial proliferation, suggesting a neuronal SASP

**Figure 5:**
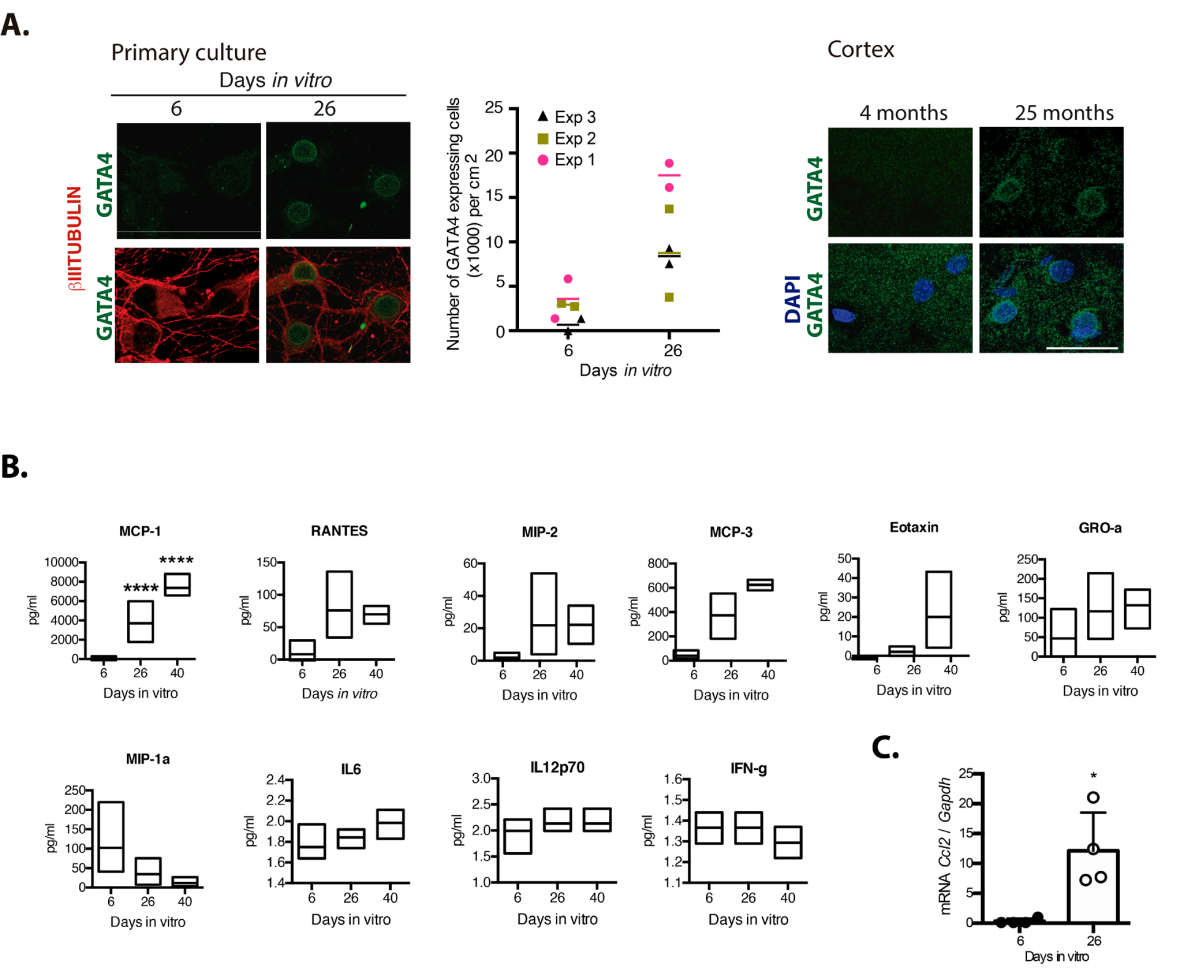
**Senescent neurons increased the expression of GATA4 and cortical cells secreted MCP-1.** (**A**) Immunofluorescence to detect GATA4 in neurons (expressing βIII-TUBULIN) in primary culture of cortical cells either incubated during the indicated days *in vitro* or in rat brains of the indicated age. Scale bars represent 25 μm. The number of cells with increased GATA4 abundance from three independent experiments, each performed in duplicate, is graphed. The mean of each experiment is represented by horizontal bars. (**B**) Quantification by multiplex immunoassay of the indicated cytokines, from conditioned media from cultures of the indicated days from three independent experiments. The maximum and minimum values are graphed. Bars indicate the mean of the three independent experiments. Data were analyzed by two-way ANOVA followed by Tukey´s multiple comparisons test analysis, only MCP-1 was significant. **** p<0.0001 relative to 6 D*IV*. (**C**) qRT-PCR from total RNA purified from cortical primary cultures during the indicated days. The relative expression of *Ccl2* mRNA was normalized with *Gapdh* mRNA. Bars represent SD. * p=0.0106 by unpaired t test two tailed. n=4.

We hypothesize that persistent senescent neurons, through the SASP, contribute to induce paracrine senescence and chronic inflammation in old brains. Therefore, we studied whether neurons would express GATA4, a transcription factor that promotes the expression of SASP factors [10]. As shown in Figure 5A, GATA4 accumulated in neuronal cells cultured for 26 D*IV*, as well in cortical neurons from old brains. To get an insight about the cytokines that senescent cortical cells could be secreting, we analyzed the presence of G-CSF, GM-CSF, IFNγ, IL-1α, IL-1β, IL-10, IL-12p70, IL-13, IL-17A, IL-2, IL-4, IL-5, IL-6, TNFα, EOTAXIN, GRO-α, IP-10, MCP-1, MCP-3, MIP-1α, MIP-2 and RANTES by a multiplex immunoassay. As shown in Figure 5B, MCP-1, RANTES, MIP-2, GRO-1, MCP-3 and EOTAXIN were more abundant in conditioned media from senescent cortical cultures, although only MCP-1 content showed a statistically significant difference at 26 and 40 D*IV* relative to 6 D*IV* cortical cultures; MIP-1a secretion was reduced in older cultures. To our surprise, IL6, a common SASP component, was barely detected and did not increase in conditioned media from senescent cells. Also, IL-12p70 and IFNγ secretion did not change along the time in culture and were secreted in a very small amount, at the threshold limit of detection. G-CSF, GM-CSF, IL-1α, IL-1β, IL-10, IL-13, IL-17A, IL-2, IL-4, IL-5, TNFα, GRO-α, IP-10 and MIP-1α were not detected at any time point. We further confirmed the induction of expression of *Ccl2* (gen coding for MCP-1) by qRT-PCR (Figre 5C).

**Figure 6:**
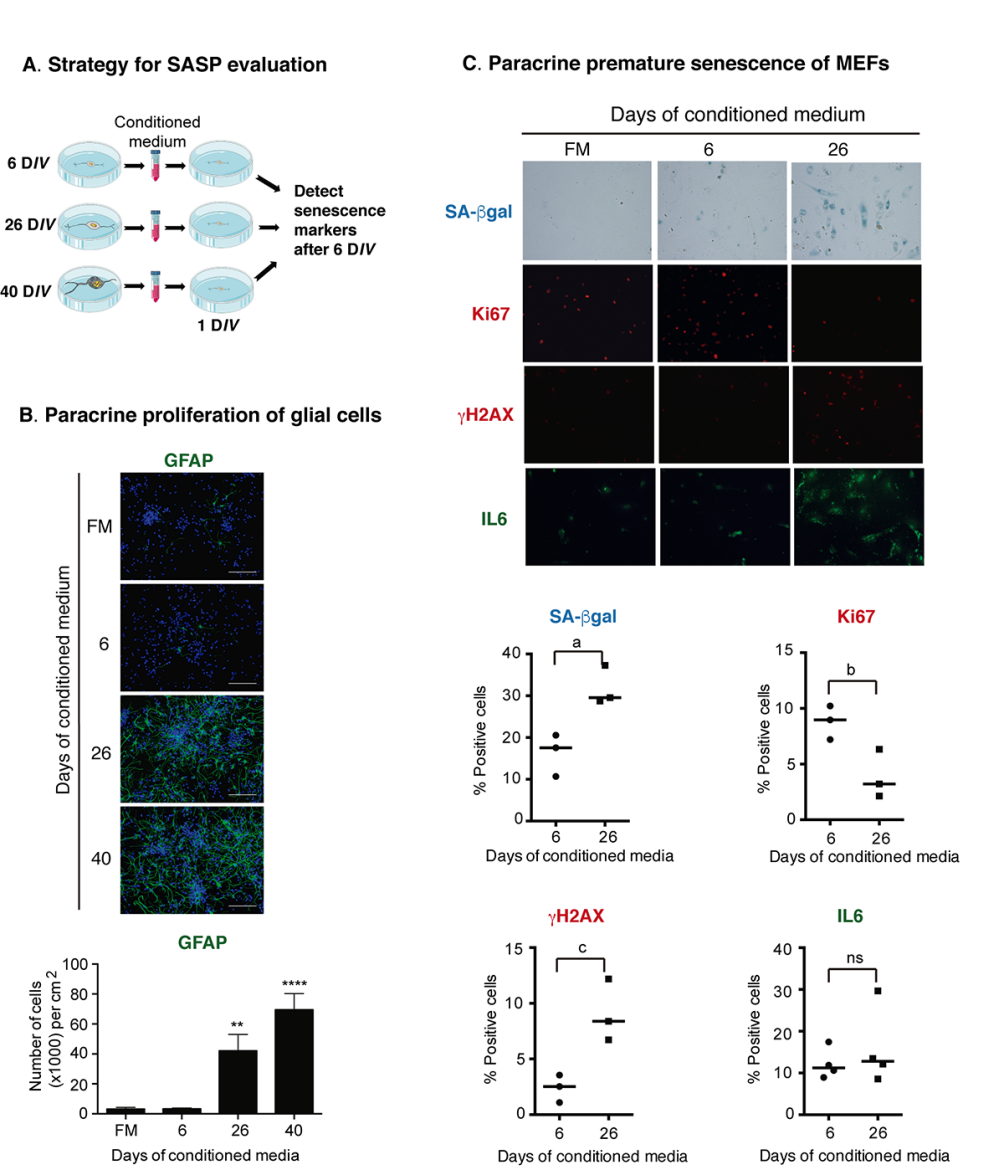
**Senescent cortical cells develop a functional SASP.** (**A**) Experimental design to evaluate the presence of secreted molecules with SASP activity from senescent cortical cells. Conditioned media was collected from cortical cells that had been incubated from 1-6 (6), 19-26 (26) or 32-40 (40) D*IV*. Either primary prenatal cortical cells or MEFs were cultured for 24 hr (1 D*IV)* before adding conditioned media; senescence markers were evaluated 6 days later. (**B**) Conditioned media from senescent cortical cells induced paracrine proliferation of glial cells in primary culture of prenatal cortical cells. Representative immunofluorescences to detect GFAP in cortical cells are shown. Cells were incubated with conditioned media collected from cortical cells that had been incubated during the indicated D*IV*. FM stands for fresh medium. Notice that conditioned media from 26 D*IV* and 40 D*IV* induced high proliferation of glial cells. Scale bars represent 500 μm. The bottom graph is a quantification of the number of GFAP expressing cells in three independent experiments, each done in duplicate. Data were analyzed by one-way ANOVA, with Dunnett´s multiple comparison test. ** p<0.01; ****p<0.0001 compared to FM. (**C**) Conditioned media from senescent cortical cells induced paracrine premature senescence in MEFs. MEFs were incubated with conditioned media collected from cortical cells that had been incubated during the indicated D*IV*. FM stands for fresh medium. Notice that senescent markers were higher in cells incubated with conditioned media from cortical cells cultured for 26 days. The bottom graphs are quantifications of the percentage of cells with blue or fluorescent signals. The signal (either blue or fluorescent) from cells incubated with 6 D*IV* conditioned media plus one standard deviation was deducted from the signal obtained from the cells treated with 26 D*IV* conditioned media. Three independent experiments, each performed in duplicate were quantified. Data were analyzed by unpaired T test. a, p=0.0175; b, p=.0.0327; c, p=0.0191.

Since MCP-1 has been shown to induce paracrine senescence [23], and GATA 4 is a mediator regulating MCP-1 expression during senescence induced by Lamin-A defects [28], we reasoned that senescent cortical cells could indeed acquire a functional SASP. To test this hypothesis, we analyzed whether conditioned media from senescent cortical cells could induce premature paracrine senescence (a schematic experimental design is shown in Figure 6A). To obtain conditioned media with accumulated secreted factors, the media was left for intervals of about a week in between fresh media changes over the cells, and it was collected at 6, 26 or 40 days of culture. Conditioned media was then added to young (1 D*IV*) prenatal cortical cells and after 6 D*IV* of treatment all the senescent markers described above were evaluated. Interestingly, conditioned media from cortical cells cultured for 26 D*IV* or 40 D*IV* induced abundant proliferation of glial cells (Figure 6B), suggesting that senescent cortical cells, potentially neurons, could indeed affect tissue organization. Nevertheless, we did not observe significant expression of senescent markers on young neurons (not shown). We reasoned that young neurons could be more resistant to paracrine senescence than mitotic cells; therefore, we tested whether the conditioned media from cortical cells cultured for 26 D*IV*, time at which most of the senescent cells are neurons, would induce paracrine senescence in mouse embryonic fibroblast (MEFs). Indeed, conditioned media from cortical cells cultured for 26 D*IV* induced key senescent features in MEFs, such as increased SA-β-gal activity; inhibition of proliferation (detected by Ki67 expression); DNA damage response identified by **γ**H2AX foci; and increased expression of IL6, although this latter was not statistical different (Figure 6C). In summary, prenatal cortical cells exposed for 6 days to conditioned media from cortical cells cultured for 26 D*IV* or 40 D*IV* did not show senescent features, whereas MEFs did.

Taken together, our results confirm that neurons acquire senescent features potentially including the SASP, interestingly before glial cells. This observation suggests that senescent neurons could affect the function of surrounding cells, such as astrocytes. Intriguingly, young neurons were unresponsive to the SASP produced by senescent cortical cells, since they did not show senescent markers when exposed to the conditioned media from cortical cultures of 26 or 40 *DIV* (not shown).

### Dysfunctional autophagy contributes to neuronal senescence

**Figure 7:**
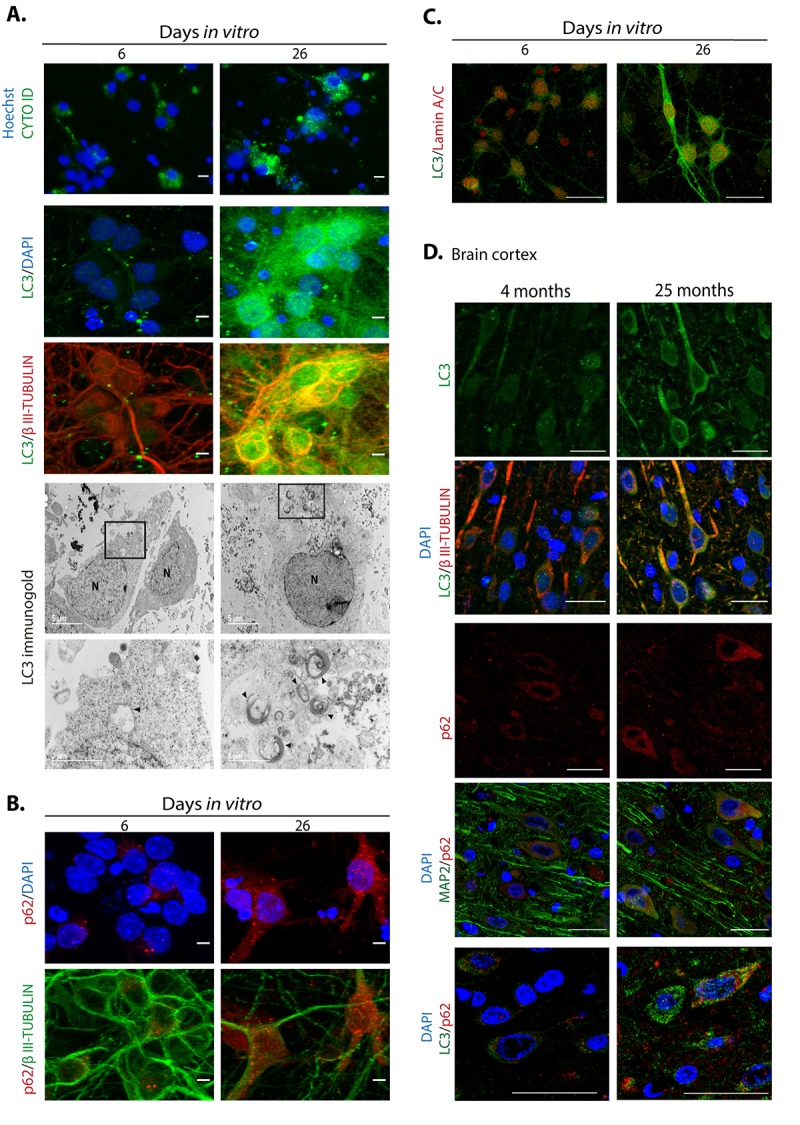
**Autophagosomes accumulate during neuronal senescence.** (**A**) Top row, autophagosomes were stained with CytoID® and nuclei with Hoechst in primary culture of cortical cells incubated during the indicated D*IV*; scale bars represent 15 μm. Middle rows, immunofluorescence to detect LC3 in neurons (expressing βIII-TUBULIN) of primary cortical cells cultivated during the indicated D*IV*. Scale bars represent 5 μm. Bottom rows, electron micrographs showing accumulation of autophagosomes in 26 D*IV* cortical cells, detected by immunogold localization of LC3 (arrow heads). Squares indicate the amplified area below. (**B**) Immunofluorescence to detect p62/SQSTM1 in cortical cells cultured during the indicated D*IV*. Nuclei were stained with DAPI. Notice that p62/SQSTM1 in neurons (expressing βIII-TUBULIN) accumulated at 26 D*IV*. Scale bars represent 5 μm. (**C**) Immunofluorescence to simultaneously detect LC3 and Lamin-A/C to observe intranuclear folds as a senescence marker, in cortical cells cultured during the indicated D*IV*. Scale bars represent 25 μm. (**D**) LC3 and p62/SQSTM1 also accumulate in cortical neurons (expressing βIII-TUBULIN or MAP2) form old rat brains. Scale bars represent 30 μm.

**Figure 8:**
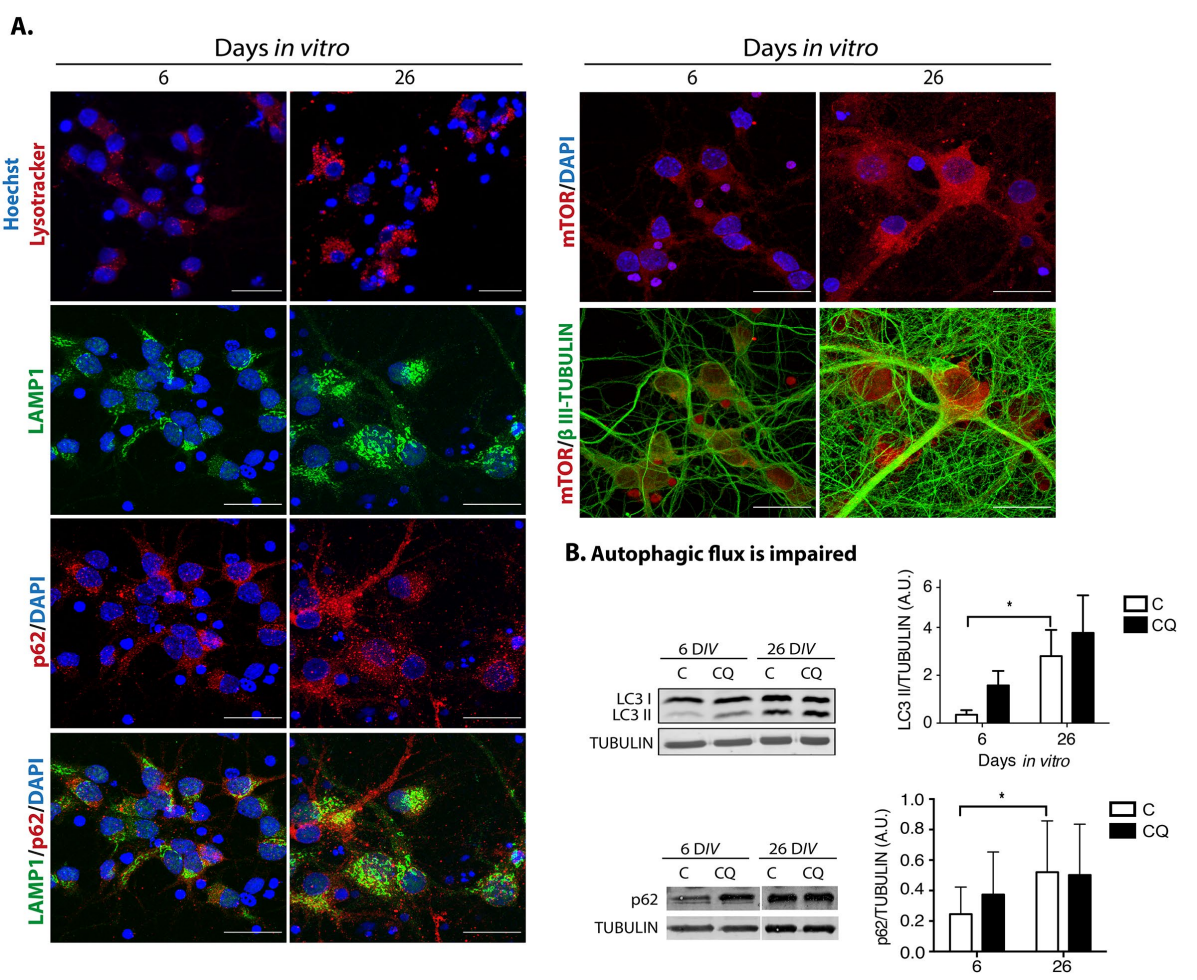
**Dysfunctional autophagy contributes to neuronal senescence.** (**A**) There was an accumulation of enlarged lysosomes and undigested p62/SQSTM1 in senescent neurons. Top row, lysosomes were detected with Lysotracker® and nuclei with Hoechst in primary culture of cortical cells incubated during the indicated D*IV*. Bottom rows, immunofluorescence to detect the indicated proteins in cortical cells cultured during 6 or 26 days. Nuclei were stained with DAPI. Notice that even though lysosomes and p62/SQSTM1 accumulated at 26 D*IV*, their intracellular distribution did not overlap. mTOR distribution did not change. Scale bars represent 25 μm. (**B**) The autophagic flux was impaired in senescent neurons. Western blot of total protein extracts from cortical cells cultured at 6 or 26 days, without (C) or with (CQ) 20 μM Chloroquine for 4 hr. Graphs represent the mean of densitometry analysis of four independent experiments. Bars represent SEM. Two-way RM ANOVA followed by Sidak´s multiple comparison test. *p<0.001.

Abundant reports indicate that dysfunctional autophagy accompanies aging, and in the brain it causes neurodegeneration [29]. Accordingly, induction of autophagy ameliorates age-related cognition deficits [30]. Therefore, we evaluated whether dysfunctional autophagy would contribute to neuronal senescence establishment, with a similar mechanism as described for senescence transition of mitotic cells. First, we analyzed in senescent neurons whether autophagic flux is reduced, reflected by accumulation of autophagosomes and proteins associated to them like LC3 and p62/SQSTM1. As shown in Figure 7A, cortical cells at 26 D*IV*, a time point when neurons showed senescent features, had more autophagosomes detected with the specific dye CytoID® and by immunofluorescent detection of LC3. We confirmed the abundance of autophagosomes in senescent neurons by electron microscopy, labeling them with immunogold localization of LC3. As the autophagic receptor p62/SQSTM1 is degraded together with the cargo, it accumulates when the autophagic flux is interrupted. We analyzed the abundance of p62/SQSTM1 by immunofluorescence (Figure 7B) and Western blot (Figure 8B), and we found that it was also accumulated in neurons at 26 D*IV*. To determine whether indeed senescent cells had more autophagosomes, we simultaneously detected LC3 and Lamin-A/C. As shown in Figure 7C, the same cells that had intranuclear folds of the nuclear envelop with Lamin-A/C at 26 D*IV* had LC3 accumulation (Figure 7C). Importantly, both LC3 and p62/SQSTM1 also accumulated in cortical neurons of old rat brains (Figure 7D). The accumulation of both autophagosomes and p62/SQSTM1 suggests dysfunctional lysosomes or impairment of the fusion of autophagosomes with lysosomes. We stained lysosomes with Lysotracker® and observed an accumulation of enlarged lysosomes in senescent cortical cells (Figure 8A), as it has been described in another model of neuronal senescence [21]. We confirmed altered lysosomal morphology by localizing the lysosomal protein LAMP1; we noticed that abundant p62/SQSTM1 puncta did not co-localize with LAMP1 at 26 D*IV* (Figure 8A), suggesting limited autolysosome maturation (*i.e.* reduced autophagosome-lysosome fusion), although further studies are needed to corroborate it.

p62/SQSTM1 accumulates when autophagy promotes the SASP through a compartmentalized structure coined TASCC (Tor-autophagy spatial coupling compartment), which is where amino acids released by autolysosomes locally activate mTORC1, facilitating the synthesis of SASP proteins. The TASCC can be distinguished by the polarized co-localization of p62/SQSTM1, mTOR and LAMP2 [11]. Therefore, we analyzed whether during neuronal senescence these proteins were also distributed in a similar polarized intracellular localization (we observed LAMP1 instead of LAMP2). As can be seen in Figure 8A, neither the distribution of p62/SQSTM1 nor mTOR indicated a compartmentalized distribution. Even though lysosomes seemed to be mainly located on one side of the cells, it might reflect only the particular morphology of this type of cells, which have the cytoplasm mostly on one side of the cell. Therefore, we found no evidence of a TASCC during neuronal senescence, indicating that autophagy dysfunction, rather than function, accompanies neuronal senescence. To verify that the observed accumulation of autophagosomes was due to an impaired autophagic flux, instead of an enhanced activation of autophagy, cortical cells were incubated in the presence of Chloroquine, an agent that neutralizes lysosomes pH and impairs autophagosomes fusion with lysosomes. As shown in Figure 8B, the presence of Chloroquine did not increase the amount of LC3-II or p62/SQSTM1 accumulation at 26 D*IV*, indicating that the autophagic flux was already diminished in senescent neurons.

**Figure 9:**
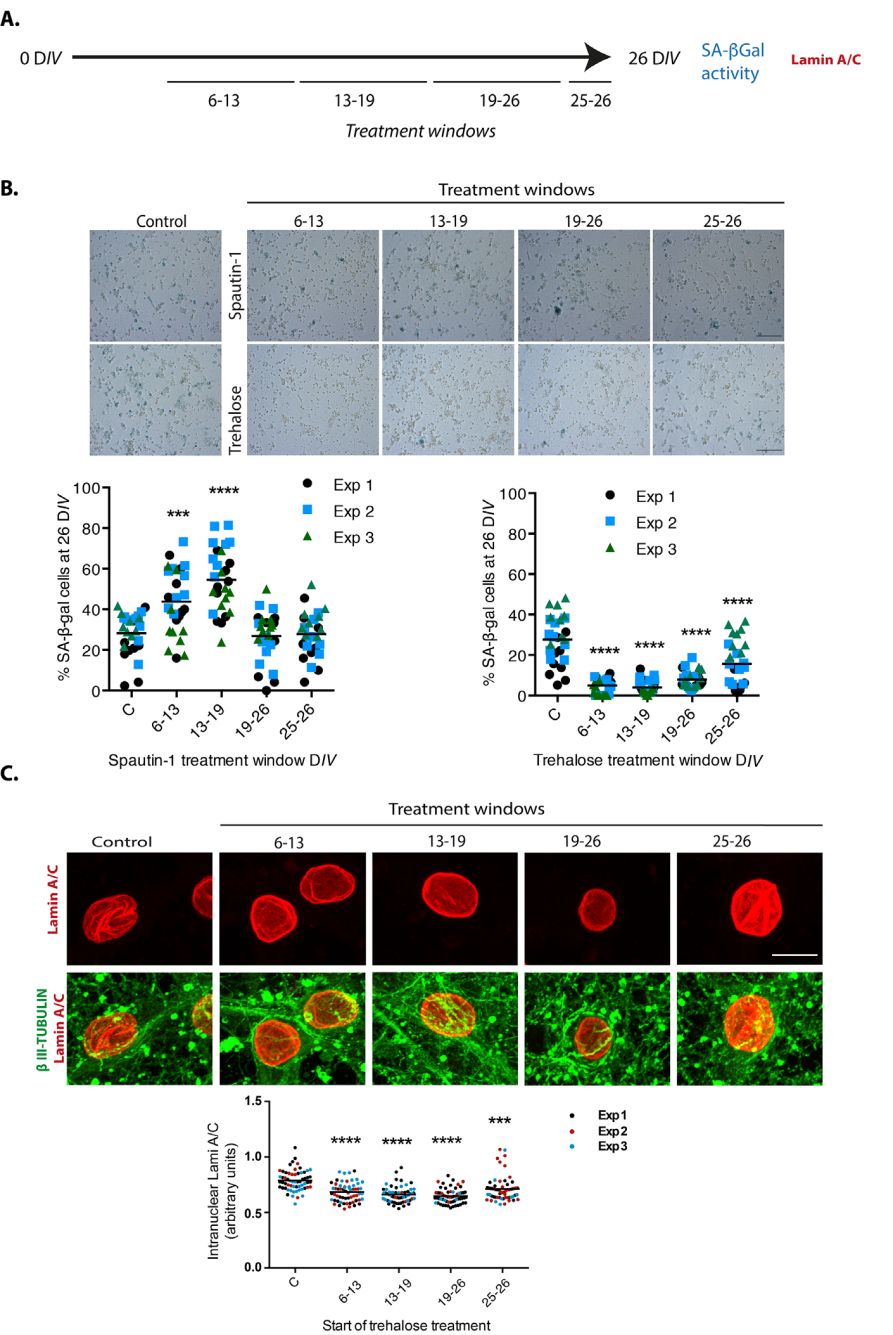
**Autophagy modulation alters cortical cells senescence.** (**A**) Experimental design. (**B**) Early inhibition of autophagy with Spautin1 increased the number of cells with SA-β-gal activity, while early induction of autophagy by adding trehalose reduced them. Spautin1 or trehalose were added during periods of several days, at the indicated time intervals in days of culture (D*IV*); after 26 D*IV* all cultures (including control with no treatment) were fixed to quantify the percentage of cells showing high SA-β-gal activity. Scale bars represent 500 μm. The bars in graphs represent the mean of each independent experiment, each done by triplicates. Three fields from each replica were scored (9 fields per experiment), each dot represent the percentage of SA-β-gal positive cells per field. Data were analyzed by two-way RM ANOVA, followed by Dunnett´s multiple comparison test. ***p<0.001 Spautin1 added during 6-13 D*IV* in comparison with control; **** p< 0.0001 Spautin1 added during 13-19 D*IV* in comparison with control, and Trehalose treatments in comparison with control. (**C**) Autophagy induction with trehalose reduced the abundance of intranuclear fold with Lamin-A/C. Trehalose were added during periods of several days, at the indicated time intervals in days of culture (D*IV*); after 26 D*IV* all cultures (including control with no treatment) were fixed to detect Lamin-A/C by immunofluorescence. Scale bar represents 5 μm. Representative images of three independent experiments are shown. At least 60 cells per treatment were quantified as described in Methods. Bars in the bottom graph represents mean. Data were analyzed by two-way RM ANOVA, followed by Dunnett´s multiple comparison test. ***p<0.001; **** p< 0.0001 with respect to control.

If a limited autophagic flux contributes to neuronal senescence, we would expect more senescent cells when autophagy is inhibited. Since basal autophagy is essential for survival, inhibition of autophagy by genetic means along the time of culture kills the neurons. Therefore, we inhibited autophagy only at distinct time windows (7 days long each) by adding Spautin-1, a molecule that indirectly induces BECN1 and PtdIns-3-kinase type 3/VPS34 (PI3KC3) degradation [31]. As shown in Figure 9B, we found that the number of cells with SA-β-gal activity increased when autophagy was inhibited during the second or third weeks of culture. When Spautin-1 was added at the fourth week of culture or later, it had no effect increasing the number of cells with SA-β-gal activity, suggesting that autophagy was already dysfunctional at this time. On the other hand, stimulating autophagy with trehalose, a disaccharide that mimics caloric restriction by preventing glucose uptake [32], reduced the number of cortical cells with SA-β-gal activity regardless of the time window of exposure, although a more noticeable protection was observed during the second and third weeks of culture. As a control to verify the function of Spautin-1 in neurons, we verified the reduction in the number of autophagosomes in cortical cells at 26 D*IV*, when we had previously observed abundant autophagosomes. We also tested that trehalose stimulates autophagy in neurons by detecting more autophagosomes in cortical cells at 6 D*IV*, which time we had observed neurons have a reduced amount of autophagosomes (Supplementary Figure 2S). To confirm that trehalose induction of autophagy indeed reduced the senescent phenotype and not just SA-β-gal activity, we repeated the experiment now immunodetecting Lamin-A/C to evaluate whether the intranuclear folds were reduced. As shown in Figure 9C, adding trehalose during one week windows statistical significantly reduced the degree of intranuclear Lamin-A/C folds. These results suggest that autophagy induction prevents senescence conversion and potentially reverts the senescent phenotype, although further experiments will be needed to address the latter.

## DISCUSSION

Accumulating evidence shows the presence of senescent cells in brains from both physiologically aged subjects and with neurodegeneration [14]. In this work we have characterized an *in vitro* model useful to study the molecular basis for neuronal senescence transition and maintenance, as well as SASP components. We demonstrate that neurons, in spite of being post-mitotic cells, acquire multiple senescent features and notably they do so before glial cells. Every senescent marker we looked at in primary culture of senescent neurons was confirmed in old rat brains (25 months old), validating this *in vitro* neuronal senescence model. We demonstrated, to our knowledge for the first time, that senescent cortical cells develop a functional SASP, secreting components able to induced paracrine senescence in MEFs and glial proliferation. Since most of the senescent cells at 26 D*IV* are neurons, we suggest the existence of a neuronal SASP, although it is still possible that the few senescent astrocytes present in the culture secrete a very potent SASP. Nevertheless, a neuronal SASP is supported by other observations, such as the induction of expression of pro-inflammatory cytokines like TNF-α and CCL2 [33]. Interestingly, dopaminergic neurons with senescent phenotype due to lack of expression of SATB1, a DNA binding protein reduced in the vulnerable region of Parkinson´s Disease patients, express several SASP components, including MCP-1 [34], the cytokine we found significantly secreted by senescent cortical cells. MCP-1 is also secreted by senescent human mesenchymal stem cells and induces paracrine senescence; according to our findings, MCP-1 expression is mediated by GATA4 [28], a transcription factor we found increased in senescent cortical neurons. Further experiments are necessary to demonstrate that indeed MCP-1 secreted by senescent cortical neurons is the molecule responsible for the induction of paracrine senescence in MEFs. To our surprise, we did no detect secretions of IL6, as it is a very common SASP component and has been found to be secreted in senescent neurons by others [22]. It is worth to note that the paracrine senescence signaling components seem to be conserved between rat and mouse, as the rat neuronal SASP was able to induce premature paracrine senescence in mouse fibroblasts (MEFs).

The finding that conditioned media from senescent cortical cells induced glial cell proliferation, suggests that persistent neuronal senescent cells could alter tissue organization. As opposed to replicative senescence, which is caused by critically short telomeres, premature senescence induced by oncogene activation initiates with hyper-proliferation, followed by a “transition phase” that signals to induce the senescent phenotype [35]. Further experiments are in progress to evaluate whether the increased proliferation of astrocytes we observed reflects a mitotic phase preceding senescence in response to the cortical SASP. Also, the finding that young neurons are resistant to undergo premature paracrine senescence in response to the SASP produced by senescent cortical cells deserves further investigation. It will be interesting to understand the molecular differences that render MEFs susceptible and prenatal cortical neurons resistant to paracrine senescence in response to the cortical SASP. Although we ought to mention that we observed in one out of six experiments an increase of young neurons with high SA-β−gal activity in response to conditioned media from senescent neurons, therefore, it is yet possible that senescent cortical cell secretes molecules able to induce neuronal paracrine senescence, but which are rather labile. We propose that persistent senescent cells in the brain would secrete molecules that induce chronic inflammation and propagate further paracrine senescence to nearby healthy cells within the brain, like astrocytes, microglia or endothelial cells, thereby spreading the senescent phenotype and thus contributing to brain aging and exacerbating neurodegeneration. In fact, chronic and low-grade inflammation (inflammaging) have been associated with neurodegeneration in aging [36,37] and cellular senescence markers have been observed in brain tissues from Alzheimer's disease patients, such as p16^INK4A^ expression, increased p38MAPK activity, and IL6 and TGFβ mRNA expression [38–41]. Actually, brain overexpression of IL6 in mice induces neurodegeneration [42]. Promising, two recent works demonstrate that eliminating senescent cells in the brain ameliorate Tau-dependent neuropathology in mice transgenic models. Bussian TJ, *et al.* did not find neurons with senescent features (looking for SA-β-gal activity and the mRNA expression of *p16*, *p19*, *p21*, *pai1* and *Il6* in an enriched population of cells expressing Cd56), and propose that senescent microglia and astrocytes induce Tau-containing neurofibrillary tangles (NFT) in neurons by an unknown mechanism [43]. Interestingly, Musi N *et al.* analyzed laser-captured microdissected cortical neurons from human brains with Alzheimer disease and compared their transcriptome signature with adjacent histopathologically normal neurons. NFT-containing neurons had a senescent gene expression profile. The same was observed in NFT-containing neurons in a Tau transgenic mouse line [44]. Therefore, it seems that all cell types in the brain are able to become senescent, but the timing of geroconversion could vary in different contexts. Since in both works senolytics ameliorated Tau-dependent neuropathology, to discover which cell type becomes senescent and how similar are senescent cells from different cell types, will be useful to design targeted therapies.

Considering that it has been widely documented that during aging autophagy becomes dysfunctional [45], that most of the interventions that improve health span and/or lifespan stimulate autophagy [46,47], and our finding that Spautin-1 treatment increased the number of senescent cortical cells, we propose that dysfunctional autophagy during aging also contributes to cellular senescence in the brain, including neurons, which in turn contributes to synaptic dysfunction. It is fundamental, therefore, to understand the mechanisms of autophagy dysfunction with ageing in the brain. Perhaps the tubular morphology of lysosomes we observed in senescent cortical neurons interferes with lysosomes fusion with autophagosomes. Therefore, treatments that promote autolysosome maturation or prevent lysosomal dysfunction could solve or prevent the onset of neuronal senescence. Interestingly, our observation that trehalose reduced the number of senescent cortical cells suggests that the neuronal senescent phenotype is reversible. Supporting this notion, addition of resveratrol, an autophagy-inducer compound, also decreases senescent hallmarks of long-term neuroglial cocultures [22]. Further experiments are necessary to test the possible reversion of the neuronal senescent phenotype.

Having a molecular understanding of neuronal senescence, new targets for pharmacological intervention could be proposed, with potential impact to prevent or reduce both physiological brain aging and neurodegeneration.

## MATERIALS AND METHODS

### Animals

Wistar rats of the indicated age in each experiment were obtained from the animal house of the Institute of Cell Physiology at the National University of Mexico (UNAM) and were housed at 22 ^0^C in 12h light/12h dark cycle. All procedures were approved by the Internal Committee of Care and Use of Laboratory Animals of the Institute (IFC-SCO51-18). Rats had *ad libitum* access to water and food.

### Cell Culture

#### Cortical primary cultures

Cortical primary cultures were prepared as described before [48], from Wistar rat embryos of 17 days of gestation. Cerebral cortices were isolated and dissociated by 1:1400 Trypsin-EDTA (15400054, INVITROGEN/GIBCO, Grand Island, NY, USA) digestion and trituration with a Pasteur pipette. Cells were suspended in Neurobasal medium (21103049, INVITROGEN/GIBCO, Grand Island, NY, USA) supplemented with 2% B27 (17504044, INVITROGEN/ GIBCO, Grand Island, NY, USA), 200 mM GlutaMAX™ Supplement (35050061, GIBCO Life Technologies, Grand Island, NY, USA) and 0.02 mg/ml Gentamicin (15710064, INVITROGEN/GIBCO, Grand Island, NY, USA). Cells were plated at a density of 1.05 x 10^5^/cm^2^ in 12-well plates precoated with Poly-L-Lysine (P1524, SIGMA-ALDRICH St. Louis, MO, USA) (0.01 mg/ml). Cells were cultured up to 40 days *in vitro* (40 DIV) at 37^o^C in a humidified, 5% CO_2_ atmosphere. Half the medium was changed every 6 days.

#### Cell viability

Cell viability was estimated by staining with LIVE/DEAD viability/cytotoxicity kit (INVITROGEN/GIBCO, Grand Island, NY, USA). Alive cells were stained with Calcein, while dead cells were stained with Ethidium homodimer-1, following manufacturer´s instructions. For every experiment, only cells with healthy nuclear morphology (non apoptotic) were quantified.

#### MEFs culture

Mouse embryonic fibroblasts were isolated from CD1 mouse embryos at E13.5 following the standard protocol [49]. MEFs were seeded at a density of 2.6x10^3^ cells/cm^2^ with Dulbecco´s Modified Eagle Medium + GlutaMAX^TM^, 10% FBS and Penicillin/Streptomycin 100 U/ml. Each experiment was performed with MEFs at cell passage 4 to avoid replicative senescence. For conditioned media experiments, one day after seeding, cells were cultured with 25% OptiMEM supplemented with GlutaMAX and 75% of conditioned media. Media and supplements were from GIBCO^®^ Life Technologies^TM^, Grand Island, NY, USA.

### SA–β-galactosidase staining

The β-galactosidase activity was analyzed following the protocols described previously [50,51]. Cells were fixed with 2% formaldehyde + 0.2% glutaraldehyde for 5 min, washed with PBS and stained with the staining solution containing: 20 mg/ml of X-gal (IB02260, IBI SCIENTIFIC, Peosta, IA, USA) in dimethylformamide, 0.2 M citric acid/sodium phosphate buffer pH=6, 100 mM potassium ferrocyanide, 5 M sodium chloride and 1 M magnesium chloride. Cells were incubated for 16 h at 37 °C. For colorimetric analysis, samples were observed in an inverted Nikon ECLIPSE Ti-U microscope, the number of positive cells was counted of at least 500 cells. For SA–β-galactosidase staining and immunofluorescence in the same samples, cells were stained for SA-β-gal as described above and then immunostained. Confocal detection of X-gal was performed as previously described [52].

### Lipofuscin accumulation

#### Autofluorescence Detection

Lipofuscin auto-fluorescence was evidenced by excitation at 450-490 nm of unstained cortical cells using an inverted Nikon ECLIPSE Ti-U microscope.

#### Sudan Black B (SBB) staining

SBB staining was performed as described [24]. 0.7 g of SBB (199664, SIGMA-ALDRICH, St. Louis, MO, USA) were dissolved in 70% ethanol, covered with Parafilm® and thoroughly stirred overnight at room temperature. Afterwards the solution was filtered (paper filter Whatman^TM^ 1001-110). Cells seeded on coverslips were fixed in 4% (wt/vol) formaldehyde/PBS for 30 min at room temperature and then washed three times at room temperature with PBS. Coverslips with fixed cells were incubated for 2 min in 70% ethanol. A drop of freshly prepared SBB was dropped on a clean slide. The coverslip with the cells was held facing down on the drop of SBB on the slide and was incubated for 10 seconds. The coverslip was carefully lifted and the SBB on the edges of the coverslip was wiped off manually from the back and along the edges of the coverslip with the help of an absorbent paper. The cells were then embedded into 50% ethanol for 1 min, transferred and washed with distilled water. The staining was observed under an inverted Nikon ECLIPSE Ti-U microscope and SBB staining was considered positive when cytoplasmic aggregates of blue-black granules were evident inside the cells. Three independent experiments, each done by quintupled replicas, were analyzed.

### Immunofluorescence

#### Primary culture

Cells were fixed with 4% paraformaldehyde for 30 min, permeabilized with PBS / 0.5% Triton for 5 min, blocked with PBS/5% BSA and incubated at 4 °C with primary antibody overnight. AlexaFluor-conjugated secondary antibodies were diluted in PBS/2%BSA (1:500, LIFE TECHNOLOGIES, Oregon, USA) and incubated for 1 h at room temperature. Nuclei were stained for 2 min with DAPI (1 μg/ml). Only cells with healthy nuclear morphology (non-apoptotic) were quantified.

#### Brain section

Wistar rat male brains 4 or 25 months old were isolated following IACUC guidelines. Rats were perfused transcardially with PBS, then with 4% paraformaldehyde. Brains were drop-fixed in 4% paraformaldehyde for 24h; for cryoprotection brains were immersed in PBS/30% sucrose for 24 h. Brain coronal sections (50 μm) from frontal cortex were mounted serially. The sections were permeabilized with PBS/ 0.3% Triton for 15 min, blocked with PBS/5% BSA for 1 hour at room temperature and incubated with primary antibody at 4 °C overnight in PBS/1% BSA. Next, sections were incubated with AlexaFluor-conjugated secondary antibodies (1:350, LIFE TECHNOLOGIES, Oregon, USA) in PBS / 2% BSA 1 h at room temperature; nuclei were stained with DAPI (1 μg/ml). To avoid lipofuscin autofluorescence slices were incubated with Sudan Black B.

The following primary antibodies were used: mouse anti class III β-TUBULIN (1:1000, ABCAM 14545, Cambridge, MA, USA), rabbit anti class III β-TUBULIN (1:1000, ABCAM 18207, Cambridge, MA, USA), rabbit anti- class III β-TUBULIN (1:500, BIOLEGEND, MRB435P-100, San Diego, CA, USA), rabbit anti-GFAP (1:1000, DAKO Z0334, Santa Clara, CA, USA), rat anti-GFAP (1:1000, INVITROGEN 13-0300, Camarillo, CA, USA), rabbit anti-p21 (1:25, ABCAM 7960 or 1:100 ABCAM 109199, Cambridge, MA, USA), mouse anti-γH2AX (1:500, ABCAM 26350, Cambridge, MA, USA), rabbit anti-LC3 (1:500, MBL PD014, Nagoya, Japan), mouse anti-p62 (1:300, ABCAM 56416, Cambridge, MA, USA), rabbit anti-LAMP1 (1:1000, SIGMA-ALDRICH L1418, St. Louis, MO, USA), rabbit anti-mTOR (1:200, CELL SIGNALING 2983, Beverly, MA, USA), rabbit anti-GATA4 (1:500, ABCAM 84593, Cambridge, MA, USA).

Images were acquired using a NIKON ECLIPSE Ti-U microscope or a confocal microscope Zeiss LSM 800. Images were processed using NIS Elements, Basic Research (NIKON INSTRUMENTS Inc ®, NY, USA) software, Version 3.13 or Fiji software.

### Immunoelectron microscopy

Cortical cells were fixed with 3% glutaraldehyde. Following fixation, dehydration was performed in an ethanol gradient: 30-40-50-60-70-80-90-100% ethanol at 4°C. Then, the cells were embedded in a LR White resin and polymerization was carried out at 50 °C. Ultrathin sections of 70-80 nm were cut from the polymer using an Ultracut-Recheirt-Jung and placed on nickel grids for immunogold assay.

The thin sections were washed twice for 2 min with deionized water and two times with PBS with 0.005% Tween20. Sections were then incubated for 30 min with the blocking solution (50 mM glycine, 0.005% Tween20, 0.01% Triton X-100 and 0.1% BSA in PBS) [53]. After blocking, sections were incubated with the primary antibody: rabbit anti-LC3 (1:500, MBL PD014, Nagoya, Japan). After rinsing three times in PBS with 0.005% Tween20, the sections were incubated overnight at 4 °C with the secondary antibody: donkey anti-rabbit 25-nm gold conjugate (Electron Microscopy Science Aurion #25708). Samples were washed three times with PBS, 0.005% Tween20 and post-fixed in 2% glutaraldehyde in PBS for 10 min. The sections were then rinsed with distilled water twice for 5 min and contrasted with 2% uranyl acetate, rinsed with water, dried and observed under a JEOL JEM 1200 EXII electron microscope.

### Immunoblotting analysis

Cells grown in the presence or absence of 20 μM Chloroquine (C-6628 SIGMA-ALDRICH, St. Louis, MO, USA) for 4 hrs were lysed in an extraction buffer consisting of 25 mM Tris, 50 mM NaCl, 2% Igepal, 0.2% SDS and 2 mg/ml protease inhibitor 18 (Complete, Roche Molecular Diagnostics, pH 7.4). Thirty micrograms of total protein were separated by SDS-PAGE and electroblotted onto polyvinylidene fluoride (PVDF-FL) membranes (Millipore). Membranes were incubated overnight with the primary antibody at 4 °C, rabbit anti-LC3 (1:1000, MBL PD014, Nagoya, Japan), rabbit anti-p62 (1:500, CELL SIGNALING 5114S, Beverly, MA, USA), mouse anti-TUBULIN (1:10000, CELL SIGNALING 3873, Beverly, MA, USA). Following three washes with TTBS secondary antibody IRDye^®^ 680RD goat anti-rabbit (925-68071, LI-COR) or IRDye^®^ 800CW goat anti-mouse (925-32210, LI-COR) was applied at 1:10,000 dilution in TTBS. Membranes were scanned and analyzed using an Odyssey® IR scanner and Odyssey® Image Studio software 5.2.5.

### Gene expression analysis

Total RNA was isolated using TRIzol™ reagent (Life Technologies), and cDNA was synthesized from 1 µg of RNA using the High Capacity cDNA Reverse Transcription Kit (Thermo Fisher Scientific #4368814). The quantitative PCR (qPCR) reaction was performed with the SYBR Green mix (Kapa SYBR® Fast Universal #KK4602) in the Rotor-Gene Qthermocycler (Qiagene, Germantown, MD, USA). All reactions were performed in quadruplicate, and the expression was normalized using the glyceraldehyde-3-phosphate dehydrogenase (*Gapdh*) mRNA. The sequences of the primers used are as follows:

*Cdkn1a* F 5′-CCGAGAACGGTGGAACTTTGAC-3′;

*Cdkn1a* R 5′-GAACACGCTCCCAGACGTAGTTG-3′

*Ccl2* (*Mcp-1*) F, 5′- ATGCAGTTAATGCCCCACTC;

*Ccl2* (*Mcp-1*) R, 5′-TTCCTTATTGGGGTCAGCAC-3′

*Gapdh* F, 5′-CTCATGACCACAGTCCATGC-3′

*Gapdh* R, 5′-TTCAGCTCTGGGATGACCTT-3′.

### Neutral Comet assay

Cells were resuspended in cold PBS at 10^3^ cells/µL density. This suspension was mixed at a 1:5 ratio with 0.75% low-melting point agarose (BIO RAD Certified™ Low Melt Agarose #1613111, Hercules, California, USA) at 37°C. About 50 to 100 µL of the mix were placed on microscope slides pre-coated with 1% normal-melting point agarose (BIO RAD Certified™ PCR Agarose #1613103, Hercules, California, USA), spread with coverslips and incubated at 4° C for 2 min and 10 min more at room temperature. The coverslips were removed and slides were covered with pre-chilled lysis solution (0.03 M EDTA, 1% SDS) for 60 min at 4°C. After that, slides were washed and covered with unwinding/electrophoresis buffer (Tris 60 mM, Acetic acid 90 mM, EDTA 2.5 mM, pH 9.0) for 60 min. Electrophoresis was performed at 25 V for 20 min. Immediately, slides were rinsed and incubated for 10 min in neutralization buffer (Tris-HCl 500 mM, pH 7.5) 3 times. Finally, DNA was stained with SYBR green (SYBR^TM^ green I Nucleic Acid Gel Stain, INVITROGEN^TM^, Eugene, Oregon, USA) 1:10000 in PBS. For each sample 50 comet images were measured, using a Nikon ECLIPSE Ti-U fluorescence microscope. The length and area of the broken DNA were measured with NIS Elements Basic Research software (NIKON INSTRUMENTS Inc ®, NY, USA)-

### SASP analysis of cortical cells

Conditioned medium was collected from neuronal cultures at 6, 26 and 40 days *in vitro* (DIV) and was frozen at -20 ᵒC until use. Concentrations (pg/mL) of G-CSF, GM-CSF, IFN gamma, IL-1α, IL-1β, IL-10, IL-12p70, IL-13, IL-17A, IL-2, IL-4, IL-5, IL-6, TNF alpha, Eotaxin, Gro α, IP-10, MCP-1, MCP-3, MIP-1α, MIP-2 and RANTES in media conditioned by cortical cells were measured by ProcartaPlex^®^ Multiplex Immunoassay (BIO RAD # 171K1002M). The conditioned medium was previously concentrated using centrifugal filter units Amicon Ultracel-3 kDa (Millipore # UFC800324) and a total of 50 μL of concentrated conditioned medium were examined following manufacturer’s instructions. Data were obtained in a Luminex Instrument and the analytes concentration was measured calibrating with a standard curve for each cytokine provided by the manufacturer.

### Cyto-ID autophagosomes detection and Lysotracker staining

The Cyto-ID (ENZO LIFE SCIENCES ENZ-51031-K200, Farmingdale, NY, USA) is an 488 nm-excitable green fluorescent reagent that specifically accumulates in autophagic vesicles. Cells were incubated in Cyto-ID (1 μl Cyto-ID/1ml cell culture medium) for 30 minutes at 37 ^o^C, 5% CO_2_ and washed prior to analysis. Lysotracker dye (DND-99 LIFE TECHNOLOGIES, Oregon, USA) was incubated for 20 min at 37ºC. Cells were analyzed by Fluorescence Nikon ECLIPSE Ti-U microscope.

### Cortical cells-derived conditioned medium collection and treatment

Conditioned medium was prepared by collecting half the medium from neuronal cultures at 6, 26 and 40 days *in vitro* (DIV) and freezing it at -20^0^C until use. Conditioned media were diluted 3:1 with fresh medium and added to cells at 1 D*IV* in 12-well plates (4x10^5^ cortical neurons cells/well; 1x10^4^ MEFs/well). For MEFs, to avoid adding unknown factors from serum, conditioned media were diluted with OptiMEM (GIBCO Life Technologies, Grand Island, NY, USA). Cells were incubated further for 6 days at 37°C and 5% CO_2_. At the end of incubation, the senescent markers were analyzed.

### Quantification

The quantifications of cells with a particular phenotype were done using NIS Elements, Basic Research (NIKON INSTRUMENTS Inc ®, NY, USA) Version 3.13 software or Fiji software. The size of the samples analyzed is indicated in every figure legend. We counted at least 100 cells in each graph shown. To quantify data that corresponds to nuclear Lamin A/C invagination (Figure 4), we exploited the observation that internal Lamin A/C invaginations increase the signal intensity of intranuclear Lamin A/C that would otherwise be in the nuclear envelope as follows: Z-Stacked maximum intensity confocal images were utilized. Border ROIs were manually selected with the brush selection tool of FIJI, the brush size corresponded to the pixel number length closest to 500 nm, roughly the nuclear envelope size (i.e. 101.41 μm x 101.41 μm 1024 pixels x 1024 pixels images required a brush of pixel size 5 and 1437 pixels x 1437 pixels images of the same metric size required a brush of pixel size 7). Border ROIs were selected manually including the most distal from the center of the nucleus signal of DAPI stained nuclei forming ring like ROIs. Central ROIs were selected to be exactly the internal part of the ring excluded from the border ROIs by using the clear outside function on the edit menu followed clicking on the internal part with the wand (tracing) tool of FIJI. The mean fluorescence intensity of these ROIs in Lamin A/C images was measured and the signal of the center was divided by the signal of the border so as to normalize for different Lamin A/C expression. Higher values correspond to more invagination.

### Statistical Analysis.

All data were analyzed and graphed with Prism 6 (GraphPad Software Inc. La Jolla, CA, USA). Specific tests were performed according to each experimental design, and are indicated in each figure.

## SUPPLEMENTARY MATERIAL

Supplementary Figures
