# Supplementary material for: Cortical neurons develop a senescence-like phenotype promoted by dysfunctional autophagy
Source: Aging (Albany NY). 2019 Aug 30;11(16):6175–98. doi: 10.18632/aging.102181 (PMC6738425; doi:10.18632/aging.102181)
Supplement: Supplementary Figures [file aging-11-102181-s001.pdf]

SUPPLEMENTARY FIGURES

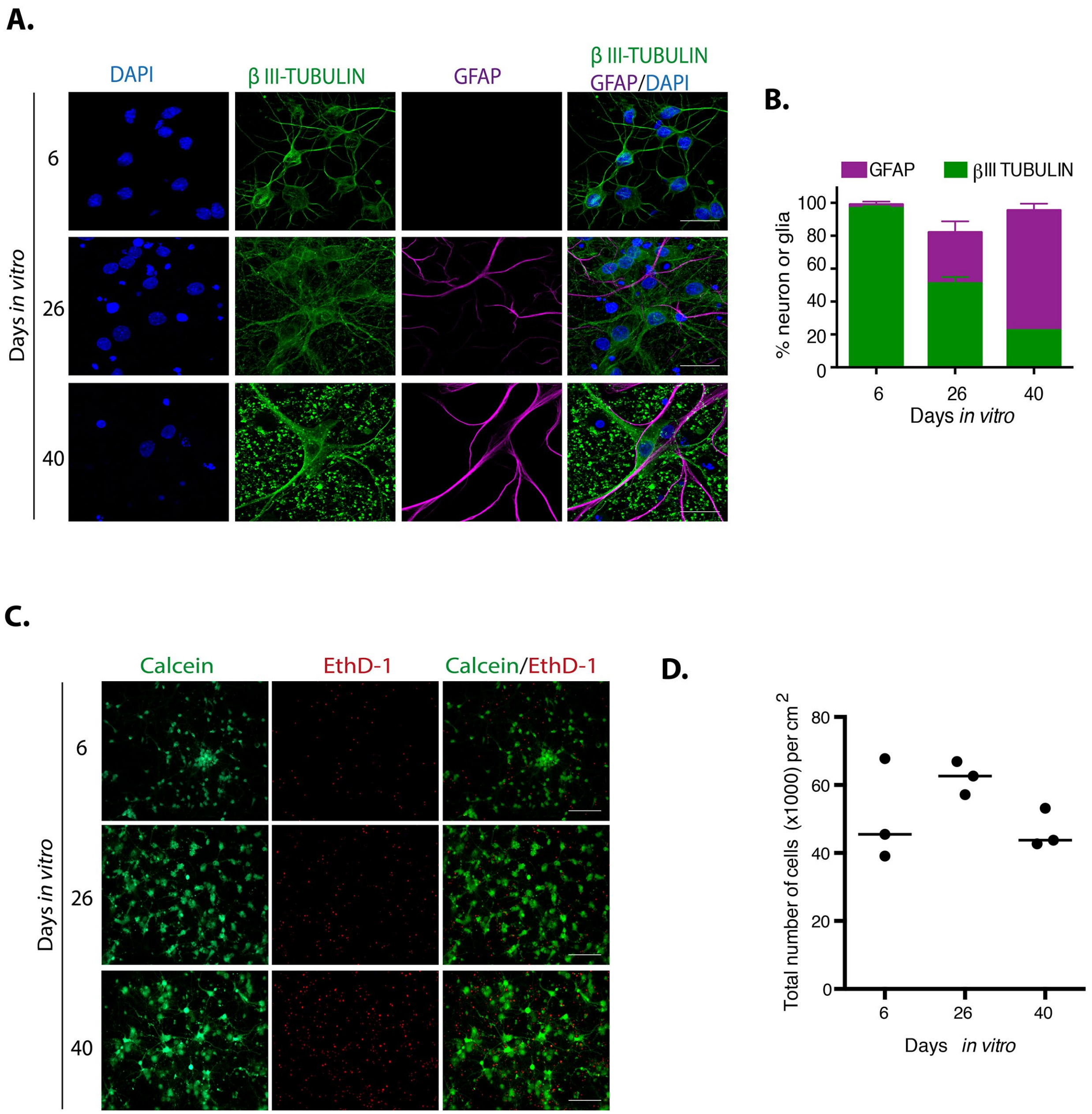

Supplementary Figure 1S. Rat prenatal cortex primary culture is viable up to 40 days *in vitro* (D*IV*). (**A**) Representative immunofluorescence used to quantify neurons (βIII-TUBULIN) and glial cells (GFAP) at 6, 26 or 40 D*IV* as indicated. Nuclei were stained with DAPI. Scale bars represent 25 µm. (**B**) The percentage of neurons or glial cells along the culture is graphed. It changes along the time of culture due to both neuronal death and glial proliferation. Bars represent SEM, n=3. (**C**) Cell viability was estimated by staining with LIVE/DEAD viability/cytotoxicity kit. Alive cells were stained with Calcein, while dead cells were stained with Ethidium homodimer-1 (EthD-1). Scale bars represent 500 µm. (**D**) The total number of cells per square cm remained similar along the culture. Each dot represents the average of three wells per experiment. Bars represent the average of three independent experiments. Although there was an increment in the average number of cells at 26 D*IV*, it was not statistically significant analyzed by two-way RM ANOVA, with Dunnett´s multiple comparison test.

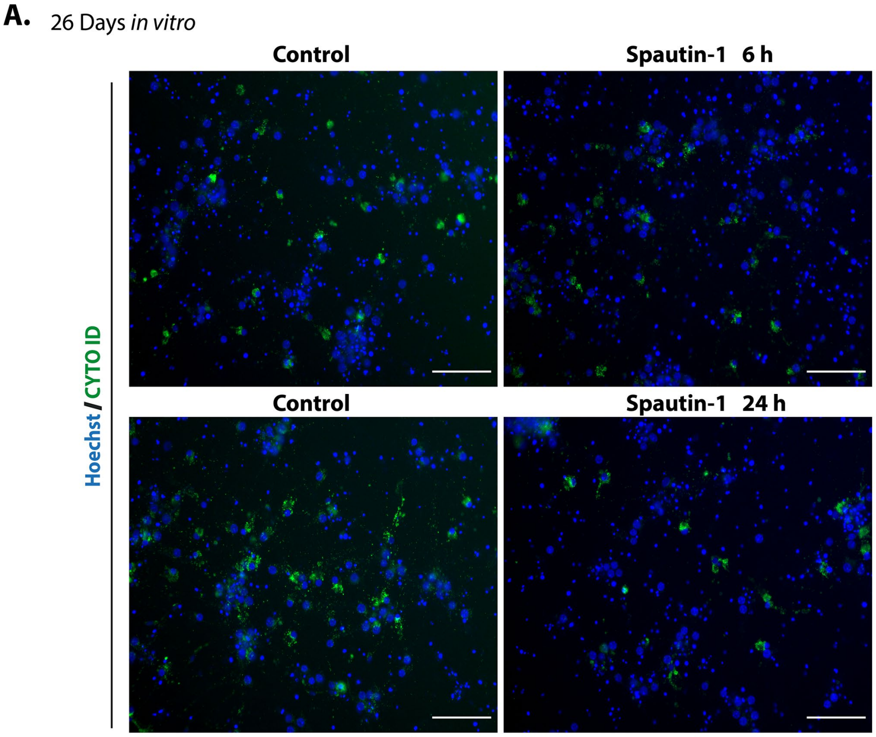

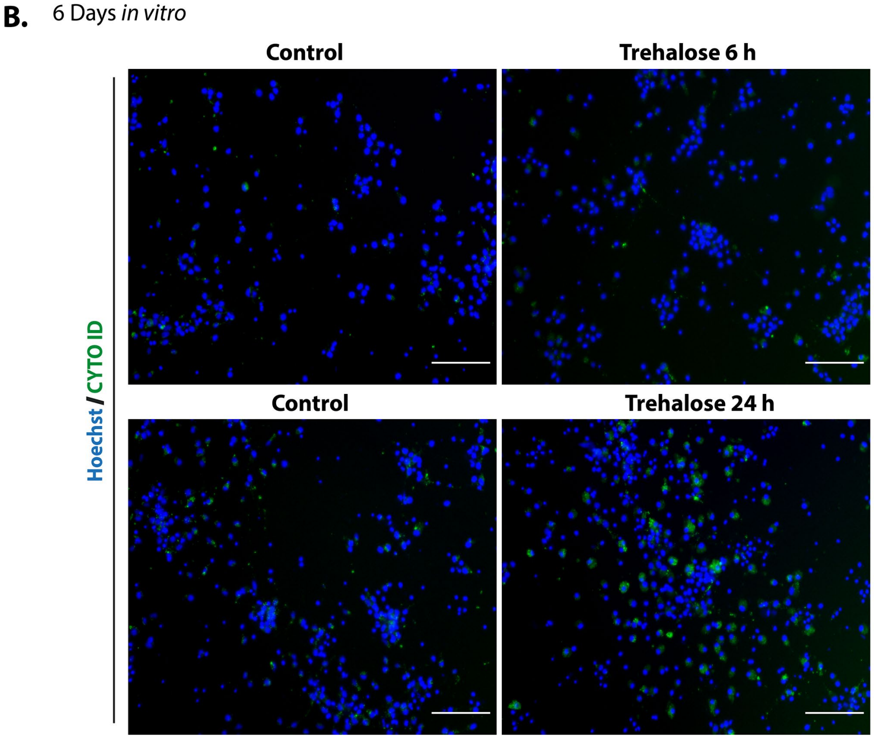

Supplementary Figure 2S. Spautin-1 inhibited autophagy and Trehalose induced autophagy on cortical primary cells. (**A**) Autophagosomes were stained with CytoID® to verify Spautin1 inhibition of autophagy on cortical cells cultured for 26 D*IV*, since at this time cells have abundant autophagosomes. Controls were treated with vehicle only. (**B**) Trehalose induction of autophagy was verified by adding it to cortical cells cultured for 6 D*IV*, since at this time cortical cells have only few autophagosomes. Controls were treated with vehicle only. Nuclei were stained with Hoechst. Scale bars represent 500 μm.
